# Starch‐Based Scaffolds for Cultivated Meat Production: Challenges and Perspectives

**DOI:** 10.1111/1541-4337.70343

**Published:** 2025-11-22

**Authors:** Cristiane Silvano Wensing, German Ayala Valencia, Edna Regina Amante, Mark Post, Silvani Verruck

**Affiliations:** ^1^ Graduate Program in Food Science Federal University of Santa Catarina (UFSC) Florianópolis Santa Catarina Brazil; ^2^ Graduate Program in Food Engineering Federal University of Santa Catarina (UFSC) Florianópolis Santa Catarina Brazil; ^3^ Graduate Program in Food Science and Technology Federal University of Pará (UFPA) Belém Pará Brazil; ^4^ Mosa Meat B.V. Maastricht the Netherlands; ^5^ Department of Physiology Maastricht University Maastricht the Netherlands

**Keywords:** food‐grade scaffolds, hydrogels, modified starches, scalability, sustainable biomaterials, tissue engineering

## Abstract

The advancement of cultured meat depends on the development of structures that combine biological compatibility, edibility, sensory acceptance, and large‐scale feasibility. In this context, starch emerges as an abundant, low‐cost, and food‐grade biopolymer, uniquely positioned for applications in food systems. Although widely studied in biomedical contexts, its specific role in cultured meat remains underexplored, despite advantages such as regulatory acceptance, consumer familiarity, and global availability. Unlike reviews focused on biomaterials for medical applications, this work centers on starch‐based structures applied to food biofabrication, considering not only the physicochemical, rheological, and structural aspects of native and modified forms, but also their performance in relation to requirements specific to the food industry. The main commercial starch sources, corn, cassava, potato, wheat, and rice, are discussed according to their relevance in global production and the availability of experimental data. The review further emphasizes processing routes such as extrusion, freeze‐drying, and 3D bioprinting, highlighting how these methods define the architecture, mechanical properties, and biofunctionality of starch matrices. The analysis balances benefits, including cost‐effectiveness, safety, and edibility, with limitations, particularly the low capacity of starch alone to promote cell adhesion. Moreover, it distinguishes results obtained from strictly biomedical studies from those that effectively contribute to food‐grade applications. Finally, industrial perspectives are discussed, including scalability, bioreactor integration, and technoeconomic aspects, positioning starch not merely as an alternative but as a strategic foundation for the design of edible scaffolds in the emerging field of food biofabrication.

**Abbreviations**
ACS: American Chemical Society
CEPEA: Centro de Estudos Avançados em Economia Aplicada
ECM: extracellular matrix
EDS: energy‐dispersive spectroscopy
ESALQ: Escola Superior de Agricultura “Luiz de Queiroz”
FAO: Food and Agriculture Organization of the United Nations
FE‐SEM: field emission scanning electron microscopy
FTIR: Fourier transform infrared spectroscopy
GPTMS: 3‐glycidyloxypropyltrimethoxysilane
MEV: microscopia eletrônica de varredura
MG63: human osteosarcoma cell line
MTT: tetrazolium salt reduction assay
PCL: polycaprolactone
PMCID: PubMed Central Identifier
PMID: PubMed Identifier
PVA: polyvinyl alcohol
SEM: scanning electron microscopy
XRD: X‐ray diffraction
μ‐CT: microcomputed tomography

## 1 Introduction

Cultivated meat has emerged as a sustainable alternative to conventional livestock farming, with potential to reduce environmental impacts and address ethical concerns associated with animal slaughter (Franziska et al. 2024; Martins et al. 2023; Roy et al. 2023). A central requirement in this field is the development of scaffolds capable of supporting three‐dimensional (3D) cell growth by ensuring oxygen and nutrient diffusion, waste removal, and structural integrity of the developing tissue (Fasciano et al. 2024).

Traditionally, scaffolds have relied on animal‐derived materials such as collagen and gelatin (Levi et al. 2023; Seah et al. 2021). However, the search for sustainable and edible alternatives has directed attention toward plant‐based polymers, which combine abundance, low cost, and regulatory acceptance (Moslemy et al. 2023; Niu et al. 2024; Seibert et al. 2025). Among these, starch stands out for its availability, renewability, and ability to form hydrogels, aerogels, and 3D‐printed structures (Roslan et al. 2016; Niu et al. 2024).

The functionality of starch depends strongly on its physicochemical properties and botanical origin. Sources such as corn, cassava, potato, rice, and wheat differ in amylose‐to‐amylopectin ratios, which influence viscosity, gelatinization behavior, and gel strength (Waterschoot et al. 2014). High‐amylose starches form more rigid gels, while amylopectin‐rich starches provide elasticity—features relevant to scaffold applications (Archana et al. 2022). Despite these advantages, native starch alone presents limitations, including insufficient mechanical strength, uncontrolled degradation, and reduced intrinsic cell adhesion (Leandro et al. 2023).

To address these drawbacks, physical, chemical, and enzymatic modifications have been applied. Enzymatic treatments, such as those involving microbial transglutaminase, or thermal processes like retrogradation, can enhance stability and improve resemblance to extracellular matrix (ECM) structures (Archana et al. 2022; Hadis et al. 2024). Chemical oxidation and blending with food‐grade proteins or polysaccharides further expand its applicability, enabling scaffolds with improved reproducibility, mechanical support, and compatibility with advanced fabrication methods, including 3D bioprinting (Miao and Benmiller 2023; Roslan et al. 2016).

This review synthesizes current knowledge on starch as a scaffold material for cultivated meat, highlighting its physicochemical, rheological, and structural properties in both native and modified forms, as well as its capacity to generate hydrogels, aerogels, and bioinks. Opportunities and persisting challenges, such as cost, scalability, and regulatory acceptance—are critically discussed, providing a framework to advance starch‐based scaffolds from laboratory research toward industrial applications. The literature was retrieved from Scopus, Web of Science, and PubMed databases, covering the period from 2010 to 2024, and includes only peer‐reviewed original and review articles addressing starch‐based scaffolds for cultivated meat or related tissue engineering contexts with relevance to food applications.

## 2 Specific Properties of Starches and Their Influence on Scaffolds

Starches exhibit a wide range of functional and physicochemical properties that directly influence their performance in scaffold fabrication. Key attributes include gelatinization temperature and enthalpy, water absorption at different temperatures, gel formation capacity, and gel strength (Maniglia et al. 2021). These properties are determined by intrinsic factors such as granule structure, amylose‐to‐amylopectin ratio, and the activity of enzymes involved in polymer synthesis during plant development (Okpalanma et al. 2024; Kumar et al. 2016).

Environmental conditions like moisture content and pH also modulate functional behaviors, particularly solubility and viscosity, which are critical in various applications (Eke‐Ejiofor et al. 2021). Swelling capacity and solubility are closely related and vary according to granule size, amylose content, the molecular architecture of amylopectin, crystallinity, and granule organization (Phogat et al. 2020).

Another important parameter is syneresis, which reflects the freeze–thaw stability of starch and its resistance to undesirable structural changes during storage and temperature fluctuations (Agvaandorj et al. 2025). Table 1 summarizes the main physicochemical properties of commonly used starches worldwide, including cassava, corn, potato, rice, and wheat, highlighting their variability and relevance for scaffold design.

**TABLE 1:** Comparison of the physicochemical parameters of native starches from cassava, corn, potato, rice, and wheat.

Parameter	Cassava starch	Corn starch	Potato starch	Rice starch	Wheat starch
**Ash content (%)**	0.61 ± 0.04 ^a^	0.1 ± 0.0 ^c^	0.1 ± 0.0 ^f^	0.24 ± 0.02 ^d^	0.19 ± 0.01 ^d^
**Particle size (µm)**	4–34 ^a^	2–30 ^e^	5–100 ^e^	1.5–8.9 ^d^	1–45 ^e^
**Moisture content (%)**	11.41 ± 0.01 ^a^	8.5 ± 0.1 ^c^	11–29 ^j^	13 ^k^	12 ^k^
**Gelatinization temperature (°C)**	66–68 ^b^	66–68 ^b^	62 ^b^	70–80 ^g^	60–70 ^g^
**Water absorption capacity (%)**	109 ± 0.5 ^a^	73.4 ± 0.40 ^d^	95 ^L^	87.39 ± 0.24 ^d^	62.9 ± 0.53 ^d^
**Viscosity (mPa s)**	2100 at 82°C ^f^	2300 at 67°C ^f^	9500 at 67°C ^f^	2500 at 71°C ^f^	2250 at 82°C ^f^
**Swelling power (g/g)**	50 (90°C) ^f^	23 (95°C) ^f^	26–49 (90°C) ^f^	17–39 (90°C) ^f^	13–25 (90°C) ^f^
**Amylose content (%)**	18–24 ^f^	23–28 ^f^	19–22 ^f^	17–21 ^f^	19–26 ^d, f^
**Amylopectin content (%)**	80–90 ^i^	75.23 ± 0.02 ^d^	70–80 ^h^	77.2 ± 0.03 ^d^	80.7 ± 0.03 ^d^
**Solubility (% at 90°C)**	13 ^f^	13–18 ^d, f^	12–17 ^f^	13–32 ^f^	11–20 ^d, f^

*Source*: Consolidated data from Hundekari and Swami (2022)^a^; Akbar et al. (2018)^b^; Bustillos‐Rodríguez et al. (2019)^c^; Alfauomy et al. (2017)^d^; Jobling (2004)^e^; Waterschoot et al. (2014)^f^; Wang et al. (2018)^g^; Zhou et al. (2022)^h^; Egbe et al. (2022)^i^; Nguyen and Trinh (2021)^j^; Mao et al. (2023)^k^; Berg et al. (2007)^l^.

Cassava starch has the highest ash content (0.61%), which may indicate a higher mineral content compared to other starches, such as corn and potato, which have significantly lower levels (0.1%). Rice and corn starches are nonallergenic due to the hypoallergenic nature of their associated proteins. Rice starch granules are the smallest known among cereal grains (Alfauomy et al. 2017). Starch granules are semicrystalline aggregates of amylose and amylopectin formed inside plant cells. While mainly composed of carbohydrates, they may also contain small amounts of lipids and proteins (Seung 2020).

Potato starch granules are larger and have the lowest gelatinization temperature compared to cassava and corn starch. Akbar et al. (2018) observed in their study a correlation between granule size and gelatinization temperature, where smaller granules tend to have higher gelatinization temperatures.

The gelatinization temperatures of wheat and rice starches are distinct, reflecting their different structural characteristics. Wheat starch typically gelatinizes between 60 and 70°C, whereas rice starch gelatinizes at a higher range, between 70 and 80°C. This variation is attributed to differences in the amylose and amylopectin content of each starch type, which affect their thermal properties and behavior during cooking (Woodbury and Mauer 2022 ).

However, such high gelatinization temperatures may preclude the use of these starches in tissue engineering strategies where cells need to be embedded in the matrix before gel formation, as the necessary heating would compromise cell viability. In these cases, cold gelatinization modifications (e.g., chemical or enzymatic changes) or incorporation of cells only after scaffold formation will be necessary to ensure biocompatibility.

Amylopectin‐rich starches, such as those from potato and cassava, generally exhibit high peak viscosity, low breakdown, and reduced setback, which reflect greater stability during cooling. On the other hand, starches with higher amylose content, such as corn starch, tend to display lower peak viscosity, accompanied by greater susceptibility to breakdown and retrogradation (Trung et al. 2017).

The swelling power of starch measures how much starch granules can absorb water and dissolve during heating, reflecting the effectiveness with which the granules incorporate liquids during gelatinization (Alfauomy et al. 2017). Additionally, a high breakdown viscosity indicates intense granule swelling, making them more vulnerable to shear forces, suggesting that rice and corn starches are particularly prone to disintegration under such conditions (Hughes et al. 2009).

Starch's ability to absorb water is influenced by various factors, including starch composition, granule surface structure, long‐range crystalline structures, short‐range ordered structures, and the presence of hydrophilic groups. Both the chain length of amylopectin and amylose content are directly associated with increased starch water absorption capacity (Zhang et al. 2023b).

To select the most suitable starch for scaffold fabrication in cultivated meat production, properties such as water absorption capacity, gelatinization temperature, and viscosity must be considered, as these characteristics directly influence scaffold–cell interactions by promoting adhesion, proliferation, and differentiation, as well as regulating nutrient diffusion and environmental stability (Good Food Institute 2022).

Cassava starch, with a high amylopectin content (80%–85%) and low‐amylose content (15%–20%), is recognized for its ability to form highly elastic gels with low retrogradation tendencies, characteristics that make it ideal for applications requiring structural stability and flexibility (Cheng et al. 2024). Additionally, high water absorption capacity, associated with its high amylopectin content (Nilusha et al. 2021), stands out as an advantage for scaffold fabrication in cultivated meat, meeting the need for a moist and stable environment during cell cultivation (Seah et al. 2021).

## 3 Scaffolding: Concept, Types, and Essential Characteristics

Scaffolds play a fundamental role in the production of cultivated meat by providing a 3D structure that supports cell adhesion, proliferation, and differentiation. To be suitable for food applications, they must be composed of edible, nontoxic, and biocompatible materials, ideally avoiding the need for surface coatings or exogenous adhesion proteins to ensure simplicity and scalability (Wei et al. 2023; Seah et al. 2021; Jones et al. 2021; Santos et al. 2023; Su et al. 2022). In addition, scaffolds should degrade in a controlled and safe manner, and mimic the ECM through adequate mechanical strength and porosity, allowing for nutrient and oxygen diffusion, characteristics essential to replicating the structure and texture of conventional meat.

Various materials have been investigated for scaffold fabrication. Animal‐derived polymers such as collagen and gelatin remain common due to their biochemical similarity to muscle ECM (Seah et al. 2021; Ahmad et al. 2021), while plant‐based alternatives like alginate, soy protein, cellulose acetate, and decellularized leaves have gained attention for their sustainability and regulatory acceptance (Wei et al. 2023; Santos et al. 2023; Seibert et al. 2025; Ben‐Arye et al. 2020). Within this group, starch stands out not only for its abundance and renewability but also for its versatility in forming hydrogels, aerogels, and printable bioinks. Unlike other natural polymers such as chitosan, alginate, or hyaluronic acid, starch combines global availability, low cost, and established GRAS status, making it uniquely positioned for food‐grade applications in cultivated meat.

**FIGURE 1:**
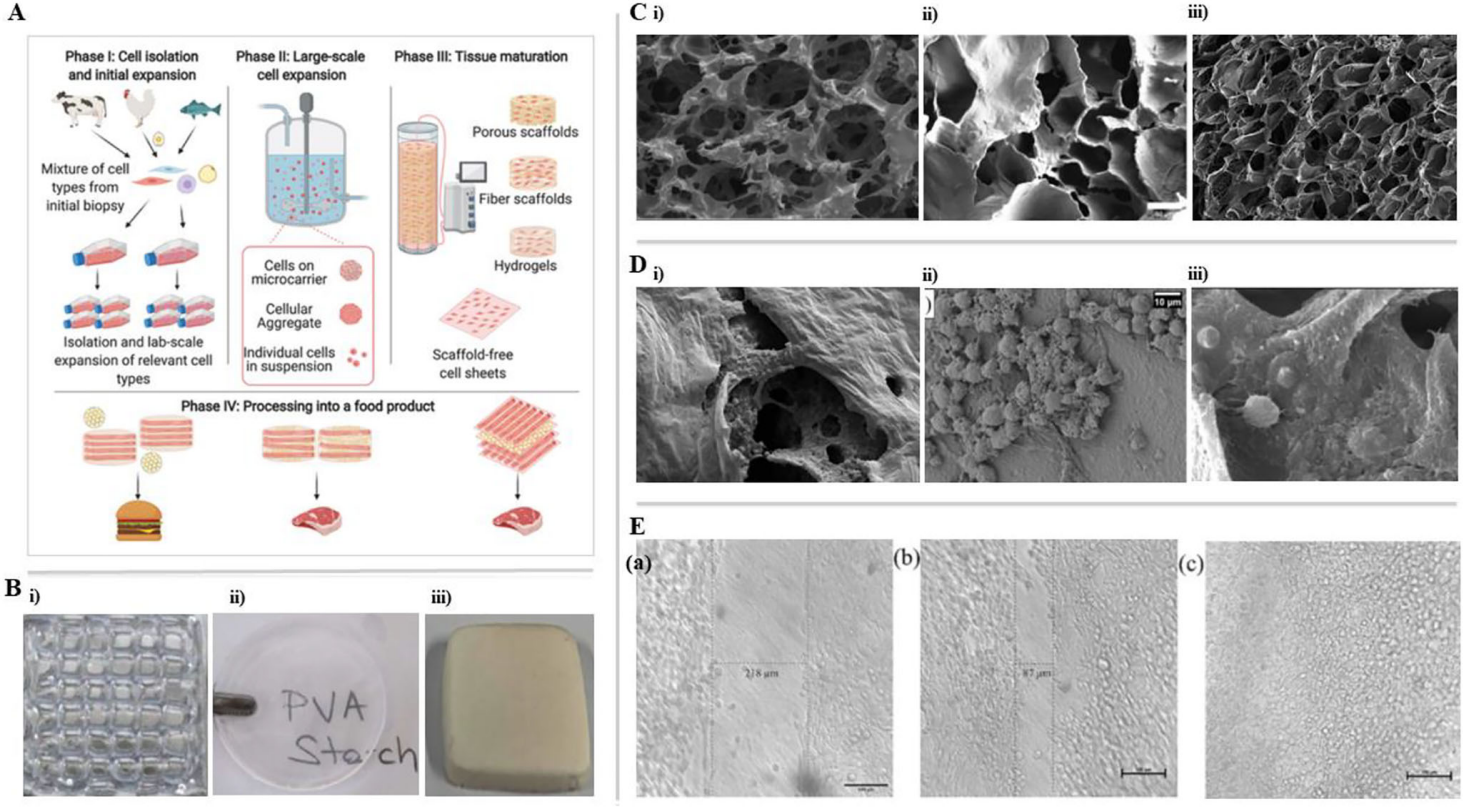
Schematic representation and morphological characterization of starch‐based scaffolds for tissue engineering and cultivated meat. (A) Schematic of the cultivated meat production process, highlighting the phases of cell isolation, expansion in bioreactors, and differentiation in scaffolds. With permission from Bomkamp et al. (2021). (B) Different types of starch‐based scaffolds, including 3D‐printed structures, membranes, and porous matrices. (i) With permission from Joseph et al. (2024) (ii) With permission from Ceylan and Demir et al. (2024). (iii) With permission from Lin et al. (2021). (C) Structural characterization by FE‐SEM of hybrid and starch‐based composite scaffolds, highlighting morphological variations. (i) With permission from Aidun et al. (2018). (ii) With permission from Joseph et al. (2024). (iii) With permission from Nourmohammadi et al. (2016). (D) Cell viability, demonstrating adhesion and morphology of different cell types in starch‐containing scaffolds. (i) With permission from Aidun et al. (2018) (ii) With permission from Ceylan and Demir (2024). (iii) With permission from Nourmohammadi et al. (2016). (E) Cell migration in three‐dimensional scaffolds over 48 h, highlighting cell‐matrix interaction. (i) With permission from Joseph et al. (2024).

Recent studies have demonstrated the potential of starch‐based scaffolds in both biomedical and food‐related applications. Figure 1 illustrates various examples of starch‐based systems. A 3D‐printed scaffold composed of arrowroot starch and gellan gum (7.5%–1% w/v) combines biocompatibility with tunable porosity for tissue regeneration. Additional architectures include corn starch membranes (Figure 1B ii) and porous scaffolds from tapioca starch and alginate (Figure 1B iii), highlighting the material's adaptability.

Microscopic analysis via FE‐SEM (Figure 1C) confirms the presence of porous and interconnected networks critical for cell infiltration. For example, starch‐GPTMS scaffolds (Figure 1C i) and arrowroot–gellan gum matrices (Figure 1C ii) displayed favorable structures for migration and proliferation. Complex blends, such as oxidized potato starch with chitosan and PCL fibers (Figure 1C iii), demonstrated controlled porosity for musculoskeletal applications.

Cellular behavior within these scaffolds was examined in Figure 1D. Mesenchymal stem cells and fibroblasts exhibited good adhesion and viability after several days, with MG‐63 cells displaying organized spreading and filopodia‐based interaction, indicating cytocompatibility (*p* < 0.05). Cell migration assays (Figure 1E) further supported scaffold functionality, showing progressive infiltration over 48 h.

Beyond biological compatibility, scaffold materials must contribute to the sensory quality of cultivated meat. One of the main challenges is replicating the elasticity and fibrous texture of conventional meat, particularly after cooking (Paredes et al. 2022; Floor et al. 2022). This has led to increased interest in scaffolds with suitable rheological properties, such as flexibility and firmness, which can mimic muscle tissue characteristics (Wang et al. 2023b; Ahmad et al. 2021).

**FIGURE 2:**
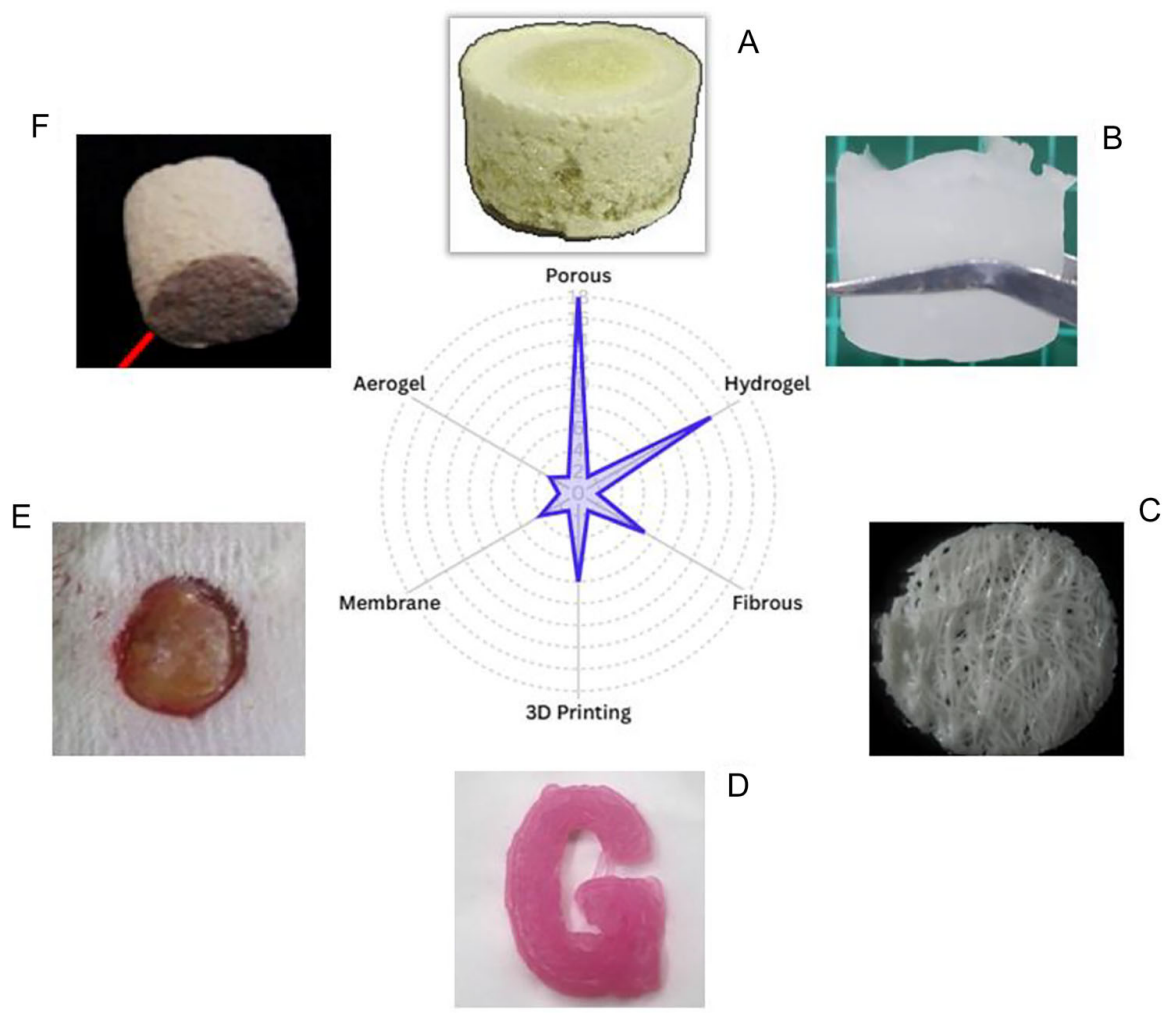
Types of starch‐based scaffolds used in tissues and their frequency in the literature. (A) Albumin and corn starch scaffold obtained without treatment using the freeze‐drying method, with permission from Prasopdee et al. (2021). (B) Starch and gelatin scaffold obtained through freezing and thawing. Available under the Creative Commons Attribution 4.0 International license. (C) Fiber mesh scaffolds, SEM micrographs, with permission from Rodrigues et al. (2012). (D) 3D‐printed structure made from wheat starch, with permission from Shi et al. (2024). (E) Membrane scaffold for wound healing on Day 1, with permission from Eskandarinia et al. (2019). (F) Aerogel scaffold formed from wheat starch, with permission from Ubeyitogullari and Cifti et al. (2016).

Preparation methods such as 3D printing, freeze‐drying, and electrospinning have enabled precise control of scaffold architecture. 3D printing and freeze‐drying, for example, are used to process materials such as hydrogels and aerogels, resulting in tunable porosity and morphology that improve cell–material interaction (Joseph et al. 2024; Shahriari et al. 2023; Jamshidi et al. 2019; Long et al. 2024), as illustrated in Figure 2, which summarizes the main scaffold formats explored in the literature.

The incorporation of components such as hydroxyapatite and cellulose improves mechanical properties like compressive modulus while maintaining biocompatibility (Soltani and Alizadeh 2023; Aidun et al. 2018). Surface properties also influence scaffold performance. Modifications in hydrophilicity, assessed through water absorption and contact angle analysis, can optimize interactions with biological systems (Shyam and Palaniappan 2024; Beh et al. 2020). While most studies have focused on applications in bone regeneration, the ability of starch‐based scaffolds to stimulate osteoblastic activity and support tissue growth suggests strong translational potential for cultivated meat systems (Nourmohammadi et al. 2016; Shahriari et al. 2023).

A notable example in the food field is the work by Niu et al. (2024), who incorporated starch nanoparticles (SNPs) into a gelatin–alginate hydrogel for 3D bioprinting. Compared to the gelatin–alginate hydrogel without SNPs (control group), the SNP‐enhanced scaffolds demonstrated improved biocompatibility and structural integrity, resulting in a 20.8% increase in satellite cell viability and a 36.1% increase in cell adhesion after 5 days of incubation. These findings underscore the potential of starch‐based materials in supporting muscle tissue formation for cultivated meat.

The architecture of starch‐based scaffolds strongly determines their biological performance. Pore size and interconnectivity regulate nutrient diffusion and cell infiltration, with larger, interconnected pores favoring vascularization and tissue formation (Zhang et al. 2022b; Jamshidi et al. 2019). Different formats meet distinct purposes: porous scaffolds enable 3D growth, fibrous structures mimic ECM to guide alignment, and membranes or mold‐based systems provide organization or selective barriers (Jin et al. 2018; Kim et al. 2023b; Mondrinos et al. 2017). Fabrication method is decisive, bioprinting offers high shape fidelity and controlled porosity, freeze‐drying yields highly interconnected pores, and starch aerogels combine ultralow density with high surface area, though requiring edible crosslinkers for stability (Wang et al. 2024b; Niu et al. 2024; García‐González et al. 2019).

Although edible scaffolds differ from biomedical implants in degradation rate and mechanical persistence, shared principles such as biocompatibility and 3D support remain relevant (Lee et al. 2024; Seah et al., 2021). Translating insights from tissue engineering to food systems underscores the potential of starch scaffolds to support scalable, ethical, and sustainable meat production (Daud et al. 2024; Takahashi et al. 2022).

Table 2 summarizes the key analytical techniques used to characterize starch‐based scaffolds. FE‐SEM provides detailed images of pore architecture, essential for assessing diffusion capacity (Couka et al. 2015). XRD, via Rietveld refinement, reveals crystallinity patterns linked to mechanical stability (Yu et al. 2020). FTIR, in combination with chemometric tools, allows prediction of chemical content and confirmation of crosslinking (Zhou et al. 2015). Together, these tools guide the optimization of scaffold composition and function.

**TABLE 2:** Applications of starch‐based scaffolds in regenerative medicine and tissue engineering.

Type	Scaffold material	Starch modification	Manufacturing method	Porosity	Application	Tested cells	Cell compatibility	Analyses
Hydrogel ^a^	Arrowroot starch extracted from *Maranta arundinacea* and gellan gum	Not modified, only chemically extracted	3D Bioprinting	_	Wound healing	Mouse fibroblasts L929	It effectively supports cell growth and proliferation. At the 30th hour, all cells have acquired an elongated morphology	FE‐SEM, XRD, EDS and FTIR; cell viability confirmed by MTT assay and cell migration studies.
Hydrogel ^b^	Soluble potato starch and citric acid	Soluble potato starch was supplied by BioBasic Inc. (Canada).	Particle leaching and lyophilization	The average pore size was measured at 130.057 ± 11.19 mm	Suitable for the attachment and growth of osteoblast‐like cells	MG63 osteoblasts	_	FTIR and XRD, SEM, EDS, MTT.
Hydrogel/ Bioink ^c^	Starch and gelatin	Starch used without modification, acquired from SRL Chemicals (Mumbai, India)	3D Bioprinting	_	Regeneration of diseased tissues, particularly for cardiac tissues	H9C2 cardiomyocytes, a cell line derived from rat cardiac tissue	Suitable mechanical properties to support cell growth and effective bioactivity with H9C2 cardiomyocytes, promoting proliferation and cell function	Rheological analyses.
Hydrogel ^d^	Starch, chitosan, polyvinyl alcohol (PVA), borax	Starch (S, 26.9% amylose content, average molecular weight), supplied by Sigma‐Aldrich (St. Louis, Missouri, USA).	Freeze–thaw method	_	Suitable for repairing exposed tissue defects	_	Excellent mechanical properties, hydrophilicity favoring interaction with body tissues, and uniform morphology, facilitating cell adhesion and proliferation	SEM, thermogravimetric analysis, FTIR spectroscopy, X‐ray diffractometry, and contact angle measurements.
Porous ^e^	Starch, aloe vera, 64S bioactive glass, and quail eggshell powder	Unspecified starch	Lyophilization	_	Bone tissue repair	MG‐63 osteoblastic cells	New bone formation and collagen fiber proliferation were observed, indicating its effectiveness in supporting cell growth and tissue repair	Antibacterial activity through inhibition zones, collagen I and Runx2 expression, and in vivo osteopontin expression after 4 weeks.
Porous ^f^	Corn starch and n‐HAp (nanohydroxyapatite)	Commercial corn starch	Solvent casting and particle leaching	53%–70% porosity.	Most suitable for bone tissue engineering applications	Osteoblastic cells	Increasing the corn starch content in the corn starch/n‐HAp scaffold results in significant porosity, optimal pore size, high crystallinity, good pore interconnection, and suitable mechanical properties	SEM, FTIR spectroscopy, X‐ray diffractometry, and compression tests.
Porous ^g^	Starch, polyvinyl alcohol (PVA), citric acid, cellulose nanofibers, and hydroxyapatite (HA) nanoparticles	Commercial starch	Unidirectional freeze‐casting and cryogenic casting followed by lyophilization	Lamellar pores aligned with widths ranging from 80 to 292 µm, suitable for bone regeneration	Most suitable for bone tissue engineering applications	Osteoblastic cells	Good cytocompatibility with osteoblastic cells and effective apatite phase mineralization due to HA nanoparticles	FTIR, FE‐SEM, and MTT.
Porous ^h^	Starch and 3‐glycidoxypropyltrimethoxysilane (GPTMS)	Starch (Mw: 342.30 g/mol) (Sigma, Saint Louis, MO, USA)	Lyophilization	Porosity decreased as a function of starch and GPTMS content	Most suitable for bone tissue engineering applications	Bone marrow mesenchymal stem cells	The growth and proliferation of bone marrow mesenchymal stem cells on the constructs confirmed the suitability of the scaffolds for bone tissue engineering applications	FE‐SEM, X‐ray diffraction.
Porous ^i^	Starch, polycaprolactone, and montmorillonite nanoclays	_	Mixture dissolution followed by salt leaching	Porosity above 70% and compressive modulus ranging from 3.3 to 5.8 MPa	Most suitable for osteoblastic cells involved in bone formation	Osteoblastic cells	_	FTIR, SEM, contact angle measurements, and compression tests.
Membrane ^j^	Gelatin, dialdehyde starch, silver nanoparticles, and curcumin	Corn starch (pharmaceutical grade, acquired from Aladdin Reagent Database Inc., Shanghai, China)	Emulsion molding	74% porosity and pore sizes of approximately 65 µm	Facilitates skin regeneration and promotes fibroblast adhesion	Mouse fibroblasts (L929)	Excellent blood and cell compatibility, significant swelling capacity (over 640%), and degradability of 18 days	Microstructure evaluation, porosity, pore size, swelling capacity, degradability, blood compatibility, and cell compatibility.
Membrane ^k^	Polyvinyl alcohol (PVA) and starch (ST)	Corn starch acquired from Sigma‐Aldrich (USA)	Lyophilization	_	Human corneal stromal regeneration, wound dressing	Mouse Embryonic Fibroblasts (MEFs)	Noncytotoxic and significantly enhanced cell attachment, migration, and proliferation	MTT and fibroblast analyses.
Fibrous ^l^	Starch and polycaprolactone	Starch, unspecified	Electrospinning	Fully interconnected porous structure	Most suitable for bone tissue engineering applications	Saos‐2 cell line, derived from human osteosarcoma	Significant cell attachment, spreading, and increased proliferation and osteoblastic activity	DNA quantification (PicoGreen), SEM, microtomography (μ‐CT), ALP quantification, and statistical analyses (Kruskal–Wallis).

*Source*: Consolidated data from ^(a)^ Joseph et al. (2024); ^(b)^ Nourmohammadi et al. (2016); ^(c)^ Shyam and Palaniappan (2024); ^(d)^ Shahriari et al.(2023); ^(e)^ Soltani and Alizadeh (2023); ^(f)^ Beh et al. (2020); ^(g)^ Mirab et al. (2018); ^(h)^ Aidun et al. (2018); ^(i)^ Jamshidi et al. (2019); ^(j)^ Long et al. (2024); ^(k)^ Ceylan and Demir (2024); ^(l)^ Gomes et al. (2008).

**Abbreviations**: ALP, alkaline phosphatase; BMSCs, bone marrow mesenchymal stem cells; CLSM, confocal laser scanning microscopy; EDS, energy‐dispersive X‐ray spectroscopy; FE‐SEM, field emission scanning electron microscopy; FTIR, Fourier transform infrared spectroscopy; H9C2, H9C2 rat cardiomyocytes; L929, mouse fibroblasts L929; MEFs, mouse embryonic fibroblasts; MG63, MG63 osteoblast‐like cells; MTT, MTT cell viability assay; μ‐CT, microcomputed tomography; PicoGreen, PicoGreen DNA quantification assay; Saos‐2, Saos‐2 human osteosarcoma cells; SEM, scanning electron microscopy; XRD, X‐ray diffraction.

Further characterization includes thermogravimetric analysis to assess thermal stability (Chen et al. 2023a), inhibition zone assays for antimicrobial properties (Calderon et al. 2019), and compression testing to evaluate mechanical performance (Zhang et al. 2022b).

Metabolic assays such as MTT, WST‐1, and alamarBlue are widely used to estimate cell viability and proliferation in scaffold‐based cultures (Präbst et al. 2017). Studies with fibroblasts, such as those by Schweinitzer et al. (2024), have demonstrated scaffold biocompatibility based on cell morphology and adhesion. Wettability, often assessed through contact angle measurements, further informs scaffold–liquid interactions and early cell attachment (Yang et al. 2024).

### 3.1 Rheological and Structural Properties of Starch‐Based Hydrogels

Composed of various biopolymers such as alginate, agarose, gellan gum, collagen, and gelatin (Piao et al. 2021), hydrogels can also incorporate biomolecules like hyaluronic acid (Larrañeta et al. 2018), expanding their applications across multiple scientific and technological fields. Hydrogels are classified based on their cross‐linking mechanism, which can be physical or chemical (Nasution et al. 2022).

Gu and Mooney (2016) highlight the nontoxicity and robust mechanical properties of natural polymer‐based hydrogels, such as those derived from polysaccharides and proteins, and their extensive applications in cell cultures. Kamoun et al. (2017) corroborate the safety of this method, particularly for functional food applications. Additionally, studies by Cui et al. (2019) and Tu et al. (2021) confirm the biocompatibility and nontoxicity of biopolymeric hydrogels, while Yu et al. (2018) and Zhang et al. (2016) report their high swelling capacity and superior mechanical properties.

Morphological structure, swelling capacity, and mechanical properties are key criteria in evaluating hydrogels as polymer matrices in various applications (Alinejad et al. 2018). These characteristics are essential in the development of functional food hydrogels, where efficacy in medium delivery and structural integrity are critical. These properties determine how hydrogels can be effectively used to encapsulate and release nutrients or bioactive agents in a controlled and efficient manner (Lu et al. 2019).

In the food sector, interest in starch‐based hydrogels is growing due to their potential use as controlled‐release systems for bioactive compounds, ingredients for 3D food printing, and bioactive films (Liu et al. 2021). Advances in research and development of these hydrogels reflect their increasing relevance and applicability in the food industry, where they offer new possibilities for innovation and product improvement (Vieira et al. 2023).

Starch is highly valued for hydrogel production due to its affordability and biocompatibility. As highlighted by Koev et al. (2020), this material is not only abundant and renewable but also biodegradable, enhancing its appeal in sustainable applications. Its physicochemical, rheological, and biochemical properties allow it to form continuous matrices, making it a promising candidate for the development of environmentally friendly and sustainable materials.

Starch‐based hydrogels are often produced using starches that undergo etherification processes (Watcharakitti et al. 2022). These modifications increase the functionality of the starch, which naturally contains an abundance of hydroxyl groups. In the case of etherified starches, ether groups, such as sodium carboxymethyl starch (CMS‐Na), which is approved for food applications, are commonly added. These starch‐based hydrogels are biodegradable and economically viable in terms of processing (Qamruzzaman et al. 2022).

Recent studies have explored alternative methods for hydrogel formation to meet the demand for more sustainable processes (Koev et al. 2020). Among them, starch oxidation by ozone and physical modifications through dry heat treatment (DHT) and high‐shear homogenization have gained attention (Maniglia et al.^2020^; Larrea‐Wachtendorff et al. 2020). These techniques offer significant advantages over conventional methods, including reduced processing times and lower associated costs.

Luthfianti et al. (2023) used a freeze–thaw method to synthesize starch hydrogels with adjustable physical properties. The results highlighted the possibility of modifying the cross‐linking density between polymer chains, thereby adjusting the swelling degree and mechanical properties of the material, making them ideal candidates for use as functional food matrices. Starch‐based hydrogels typically have weaker crosslinking than synthetic polymers due to the presence of long glucose chains in their structure. If necessary, to strengthen the hydrogel matrix and improve its crosslinking, adaptations in the synthesis process can be applied. These adaptations may include combining starch with other polymers, using a retrogradation process, and applying acid hydrolysis to break down glucose chains (Arisma 2017).

Amylose, the predominantly linear component of starch, plays a crucial role in hydrogel characteristics. Hydrogels with high‐amylose concentration tend to have greater mechanical strength and lower water absorption capacity, while those with low‐amylose content are more flexible and less elastic. These differences in amylose composition directly impact the microstructure of hydrogels (Gao et al. 2022; Biduski et al. 2018).

Therefore, for the development of hydrogels capable of mimicking muscle tissue, it is essential to consider the mechanical properties of the material to ensure its functionality and biocompatibility (Zhao et al. 2020). Muscle tissue exhibits viscoelastic behavior, allowing temporary deformations with recovery after the removal of applied force (Bin and Cao 2023).

Skeletal muscle tissue has an elastic modulus ranging from 10 to 100 kPa, depending on the state of contraction or relaxation (Yin et al. 2018). Studies by Tomasch et al. (2022) indicate that hydrogels with an elastic modulus between 5.1 and 20.6 kPa promote greater cell proliferation. Additionally, the shear force of raw meat is strongly correlated with collagen content, with pork, at 18°C, presenting a shear force of 22.80 ± 0.75 N (Põldvere et al. 2016).

The viscoelastic properties of minced beef vary according to fat content, with an increase in storage and loss moduli as fat content rises (Cevik and Icier 2020 ). Myofibrillar proteins (MPs) also exhibit distinct rheological properties depending on the meat source: MPs from pork show higher gel strength, while those from fish demonstrate the weakest (Haifeng et al. 2021).

To replicate this behavior, hydrogels need to have high elasticity, allowing significant deformations without permanent damage, similar to muscle fibers (Bin and Cao 2023). This characteristic is essential for hydrogels to withstand contraction–relaxation cycles without compromising their structural integrity (Shi et al. 2023). However, native starch hydrogels alone often lack the mechanical resilience required for such dynamic conditions (Su et al. 2024c). Therefore, to achieve the viscoelastic performance needed for mimicking muscle tissue, starch is typically combined with other polymers (such as proteins or polysaccharide gums) or chemically modified to enhance its elasticity, strength, and structural integrity.

#### 3.1.1 Biological Relevance of Physicochemical Properties

Despite progress in the development of starch‐based scaffolds, it is still not fully understood how physicochemical properties such as surface charge, hydrophilicity, mechanical resistance, and degradation rate act together to influence cellular responses in cultivated meat. These factors are interrelated and directly affect protein adsorption, cell adhesion, proliferation, and differentiation, but their correlation in starch‐based systems remains poorly investigated (Pashkuleva et al. 2004, 2009; Nieuwenhove et al. 2015; Niu et al. 2024; Kamel et al. 2025).

Surface charge and hydrophilicity are among the first variables to modulate scaffold bioactivity. Positively charged or polar surfaces, achieved through nanoparticle incorporation, chemical oxidation, or protein coatings, tend to promote cell adhesion and proliferation. Neutral or slightly negative surfaces can sustain growth but often require additional modifications to improve performance (Wang et al. 2024b; Feng et al. 2024; Lee et al. 2024; Ikuse et al. 2024). Increasing hydrophilicity through plasma treatment, nanoparticle incorporation, or bioactive coatings has also been associated with enhanced proliferation and differentiation (Niu et al. 2024; You et al. 2025). However, excessive wettability may compromise scaffold stability, accelerating degradation or causing swelling, and is rarely effective without parallel optimization of mechanical and structural properties (Kamel et al. 2025; Tang et al. 2024; Fasciano et al. 2024).

The degradation rate is equally critical, as it defines scaffold stability in aqueous environments and the maintenance of mechanical properties. When properly controlled, degradation allows the scaffold to support cell proliferation while gradually breaking down. In C2C12 myoblast models, for instance, a 2.81‐fold increase in cell number was observed after 7 days in 3D‐printed starch‐based scaffolds, a result attributed to the balance between initial stability and progressive degradation (Wang et al. 2024b). This behavior also supports differentiation, as scaffolds that preserve mechanical support while degrading gradually provide a suitable microenvironment for the upregulation of myogenic proteins and the formation of multinucleated myofibers (Su et al. 2022; Jeong et al. 2024b). Well‐regulated degradation rates not only sustain cell viability but also contribute to generating tissues with structural and nutritional properties closer to conventional meat (Lee et al. 2024).

Mechanical resistance plays a dual role, providing structural support and acting as a biomechanical cue. Compression moduli tuned to approximate native muscle stiffness have been shown to favor myogenic differentiation and maturation (Feng et al. 2024; Wu et al. 2024; Wang et al. 2024b). Cross‐linking strategies and composite formulations enable adjustment of these properties according to cell type and culture duration (Fang et al. 2024; Tahir and Floreani 2022). Conversely, scaffolds that are too rigid or brittle may hinder proliferation and alignment, highlighting the importance of mechanical balance (Shyam and Palaniappan 2024; Jung et al. 2025).

Taken together, the studies demonstrate that the biological performance of starch‐based scaffolds results from the combined effects of surface charge, hydrophilicity, mechanical strength, and degradation. These factors do not act in isolation: while charge and wettability regulate protein adsorption and early cell–material interactions, stiffness, and degradation rate determine whether proliferation and differentiation can be sustained over the course of culture.

### 3.2 Properties and Applications of Starch‐Based Aerogels

An aerogel is a solid, porous, ultralight, and translucent network formed by removing the liquid from its pores while preserving the original structure. Produced from various precursors, including biopolymers, these materials combine unique characteristics such as high surface area and low density, making them promising for a wide range of applications (García‐González et al. 2019).

Among biopolymeric aerogels, those based on starch stand out due to their environmentally friendly production, employing processes such as gelatinization, retrogradation, solvent exchange, and supercritical CO_2_ drying. These materials have broad applications in bioactive compound encapsulation and release, tissue engineering, biomaterials, packaging, thermal insulation, and the development of novel functional products (Fan 2019).

In addition, other biopolymers such as cellulose, hemicellulose, pectin, alginate, chitosan, and chitin are also widely used in aerogel production due to their surface properties, which make them ideal for therapeutic and pharmaceutical systems (Ahmad et al. 2020).

The combination of high porosity, ultralow density, and extensive specific surface area makes biopolymeric aerogels ideal candidates for scaffolds in tissue engineering and regenerative medicine. They have already been successfully applied in the regeneration of skin, cartilage, bones, heart valves, and blood vessels, promoting cell growth supported by growth factors. These 3D scaffolds not only support tissue regeneration and wound healing but also allow for the controlled release of therapeutic agents (Bashir Yahya et al. 2021; Jeong et al. 2024b).

Various methods have been employed in the fabrication of aerogel scaffolds, ranging from early techniques such as freeze‐drying and gas foaming to modern approaches such as 3D printing and rapid prototyping, which enable the creation of precise shapes tailored to the specific needs of different tissues (Yahya et al. 2020; Gopinath et al. 2020). For instance, studies have shown that pores ranging from 20 to 160 µm favor cell growth, while larger pores, between 250 and 500 µm, are more effective for cartilage repair (Mahumane et al. 2018; Ghafari et al. 2019; Lien et al. 2009).

Recent research has reinforced the potential of starch‐based aerogels in various applications. Milovanovic et al. (2024) produced corn starch aerogels with 86% porosity and a surface area of 225 m^2^/g, demonstrating controlled release of bioactive compounds for up to 3 days. Another study by Lozano‐Pineda et al. (2022) employed vacuum drying to fabricate aerogels from potato and rice starches, revealing homogeneous porous structures with high economic feasibility.

Ali and Ciftci (2016) developed biodegradable wheat starch aerogels, highlighting their high surface area, low density, and thermal stability, thus expanding their applications in bioactive delivery systems. Starch aerogels also exhibit superior biodegradability, as demonstrated by Soorbaghi et al. (2019). Their greater specific surface area facilitates cell adhesion. This also contributes to accelerated degradation, making them a sustainable alternative to synthetic materials.

### 3.3 3D printing of Starch‐Based Scaffolds

3D printing has been increasingly explored for the production of starch‐based scaffolds, as it offers control over architecture, improved reproducibility, and the possibility of incorporating different functions into a single structure (Jovic et al. 2019; Fasciano et al. 2024; Mujumdar 2024). Compared with traditional techniques such as freeze‐drying or aerogel formation, extrusion‐based printing allows the adjustment of parameters such as pore size, shape fidelity, and internal geometry, which are directly related to cell adhesion, nutrient diffusion, and tissue development in cultivated meat (Wang et al. 2024b; Rong et al. 2023; Niu et al. 2024).

Recent studies emphasize the need to optimize the rheological properties of starch‐based bioinks to achieve proper extrudability and shape retention after deposition. The incorporation of hydrocolloids, proteins, or nanoparticles has shown positive effects on viscosity, prevention of phase separation, and structural stability during printing (Liu et al. 2023; Rong et al. 2023; Wang et al. 2024b). These adjustments also influence mechanical performance: scaffolds with compressive modulus in the range of 200–300 Pa have been considered suitable for muscle cell growth, while starch–protein composites improved structural strength and enabled the fabrication of more complex geometries (Niu et al. 2024; Xian et al. 2024).

Regarding biocompatibility, starch‐based bioinks have shown favorable outcomes, supporting cellular interactions in different contexts, from fibroblasts and myoblasts to keratinocytes and other cell types with biomedical applications (Jovic et al. 2019; Hussain et al. 2021; Fasciano et al. 2024). For meat cultivation, starch hydrogels combined with nanoparticles stood out for promoting myoblast proliferation and differentiation, contributing to the formation of thicker myofibrils and tissues with improved functionality (Niu et al. 2024). Another approach has been the use of flat starch–alginate films, which may accelerate early cell adhesion and reduce processing steps (Lee et al. 2024).

From a structural perspective, 3D printing enables the development of scaffolds with controlled porosity (80–120 µm), associated with improved nutrient transport and myogenic differentiation (Wang et al. 2024b). Tailored structures not only reproduce features of the ECM but also allow adjustment of digestion rates, water stability, and sensory attributes, bringing material science closer to food design (Rong et al. 2023; Mujumdar 2024). Furthermore, some studies highlight that starch‐based bioinks can contribute to enhanced nutritional and sensory profiles, including greater moisture retention, thermal stability, and modulation of flavor compound release (Mirzapour‐Kouhdasht et al. 2024; Liu et al. 2024b).

Despite these advances, the industrial application of starch‐based 3D printed scaffolds still faces limitations. Reproducibility, production costs, and compliance with food safety requirements are aspects that require further attention. Many studies employ biomedical‐grade polymers or additives, but the adaptation to edible materials depends on the development of safe composites and environmentally friendly modification techniques (Rashwan et al. 2024; Kaith et al. 2024). Even so, the use of starch‐based 3D‐printed scaffolds in bioreactor systems is a promising perspective, as it combines geometric control, scalability, and alignment with the sensory and nutritional expectations of cultivated meat.

## 4 Evaluation of Starch‐Based Scaffolds With Different Cell Types

### 4.1 Biomedical Studies and Contributions to Tissue Engineering

Much of the current knowledge on starch‐based scaffolds comes from biomedical research, including applications with fibroblasts, osteoblasts, and mesenchymal stem cells. Studies have shown that amylopectin‐rich starches favor dense networks that support fibroblast adhesion (Cornejo‐Ramírez et al. 2018; Kobayashi and Tovar‐Carrillo 2015), while combinations with polymers such as hyaluronic acid, cellulose nanofibers, or hydroxyapatite improve mechanical stability and cytocompatibility in osteogenic and dermal models (Mirab et al. 2018; Beh et al. 2020; Eskandarina et al. 2019). Others reported enhanced proliferation when starch was chemically modified or combined with polymers like gelatin, chitosan, or PVA (Aidun et al. 2018; Caldera‐Villalobos et al. 2023; Ceylan and Demir 2024). Overall, these findings confirm a consistent trend: starch alone is insufficient to sustain adhesion, proliferation, and function, requiring modification or combination with other bioactive components.

However, many of these additives, such as hydroxyapatite, borax, or silver nanoparticles, are incompatible with food applications. For cultivated meat, the focus must shift to food‐grade biopolymers such as alginate, carrageenan, and pectin, already approved as GRAS materials, and plant‐derived proteins like soy or pea, which combine safety with biocompatibility (Liao et al. 2021; Martău et al. 2019). This perspective bridges biomedical insights with the unique requirements of food‐safe scaffold development.

### 4.2 Studies Relevant to Cultivated Meat Applications

Although starch‐based scaffolds were initially investigated in biomedical contexts, recent studies have increasingly redirected their application toward cultivated meat, both in muscle and adipose tissue models. Overall, starch has not been used as a stand‐alone material but rather as part of hybrid systems, combined with biopolymers (alginate, gelatin, bacterial cellulose, soy protein) or nanoparticles to achieve the necessary mechanical, architectural, and biological performance.

In muscle models, several scaffold formats have been explored. Lee et al. (2024) produced alginate, corn starch films that exhibited low cytotoxicity and high biocompatibility with bovine myoblasts, leading to a strong upregulation of myogenic markers PAX7 (16.5–22.8‐fold from Days 1 to 3) and MHC (32.7–41.8‐fold at Day 7). Wang et al. (2024b) developed a 3D‐printed starch‐based gel reinforced with calcium carbonate nanoparticles and glucono‐delta‐lactone, achieving controlled porosity (80–120 µm) and a compressive modulus of 246.8 Pa.

This scaffold supported C2C12 proliferation, with a 2.81‐fold increase in cell number after 7 days, along with a fusion index of 57% and a maturation index of 34.6%. Niu et al. (2024) incorporated SNPs into gelatin–alginate bioinks, which enhanced fish satellite cell proliferation (+20.8%) and adhesion (+36.1%) within 5 days, while higher nanoparticle concentrations promoted fusion and the formation of thicker myofibrils.

Similarly, Lin et al. (2021) used a freeze‐gelation method to produce porous alginate–tapioca starch scaffolds that showed good biocompatibility and supported myogenic differentiation in vitro. More recently, Kamel et al. (2025) developed alginate hydrogels enriched with tapioca starch and soy protein, with or without xanthan gum, which maintained integrity without cytotoxicity and supported bovine myoblast proliferation with sustained expression of PAX7 and desmin. Earlier work by Lv et al. (2016b) demonstrated that bacterial cellulose–potato starch composites provided tunable porosity, enabling muscle cell infiltration and improved tissue organization in both in vitro and in vivo models.

Starch has also been investigated for adipose tissue formation. Henriksson et al. (2017) employed starch–nanocellulose scaffolds in 3D bioprinting to support adipocyte maturation and lipid accumulation. Similarly, Xie et al. (2020) reported that collagen hydrogels crosslinked with oxidized starch enhanced the proliferation of adipose‐derived stem cells, highlighting starch's potential to support fat tissue development.

Beyond rheological and structural properties, the role of starch in topographical modulation of 3D‐printed scaffolds has also been reported. Rao et al. (2023) and Gasparotto et al. (2022) observed that starch contributed to the formation of surface lines and ridges that facilitated the alignment and differentiation of myogenic cells. These findings suggest that not only porosity and stiffness but also microtopography play a decisive role in guiding cell behavior, reinforcing the need for hybrid systems with well‐defined 3D architectures.

Manufacturing methods strongly influence scaffold outcomes. Thin‐film casting, as in Lee et al. (2024), reduced processing time compared with thick gels while supporting early cell adhesion. Freeze gelation, applied by Lin et al. (2021), produced homogeneous porous scaffolds suitable for muscle differentiation. Additive manufacturing techniques such as 3D printing and bioprinting allowed fine architectural control: Wang et al. (2024b) optimized geometry and mechanical stability using CaCO_3_ nanoparticles, while Niu et al. (2024) improved gel viscosity and promoted higher fusion rates with SNPs.

Alternative approaches, such as modifying bacterial cellulose self‐assembly with potato starch, generated bilayer scaffolds with tunable porosity and improved cellular organization (Lv et al. 2016b). Additionally, functional additives including CaCO_3_ nanoparticles, xanthan gum, and soy protein have been used to tailor rheological behavior, enhance stability, and improve scaffold biofunctionality (Wang et al. 2024b; Kamel et al. 2025).

Taken together, these studies demonstrate that starch contributes significantly to the structural, topographical, and biological performance of scaffolds for cultivated meat. Its presence improves printability, pore control, and cell differentiation across formats ranging from films and porous matrices to 3D‐printed gels. However, no study to date has employed starch in isolation; in all cases, it has been incorporated into hybrid scaffolds. This recurring limitation underscores that while starch adds valuable functionality, its practical application in cultivated meat depends on carefully designed formulations that ensure mechanical stability, biocompatibility, and reproducibility (Bomkamp et al. 2021).

While these studies demonstrate the biological potential of starch‐based scaffolds, their translation to food applications requires additional considerations regarding edibility, safety, and regulatory compliance, which are addressed in Section 4.3.

### 4.3 Food‐Grade Applicability of Starch‐Based Scaffolds

The adoption of starch‐based scaffolds for cultivated meat requires the use of food‐compatible materials and processes. Unlike biomedical formulations, which often incorporate hydroxyapatite, PVA, borax, or silver nanoparticles, the food‐grade approach prioritizes hydrocolloids and biopolymers recognized as safe (GRAS), widely used in the food industry and with a history of biocompatibility and biodegradability. Examples include alginate, carrageenan, and pectin, as well as natural polymers such as chitosan and plant‐derived proteins (soy, pea, zein) (Liao et al. 2021; Martău et al. 2019; Seibert et al. 2025).

Recent studies describe routes to structure starch‐based scaffolds without relying on nonedible chemical crosslinkers. For instance, ionic/acidic gelation with CaCO_3_/GDL has been applied to 3D‐printed starch bioinks, resulting in enhanced water stability and support for C2C12 proliferation and differentiation (Wang et al. 2024b). Similarly, protein‐based matrices crosslinked with microbial transglutaminase and subjected to physical post‐treatments (such as steam annealing) have shown potential as edible, cell‐compatible scaffolds (Wei et al. 2023). Other approaches have demonstrated the feasibility of food polysaccharide hydrogels (agarose, gellan) enriched with plant proteins, which exhibited noncytotoxic leachates and tunable gel stability for cell encapsulation (Wollschläger et al. 2022). In addition, decellularized plant scaffolds have been adapted for food applications by replacing nonedible solvents with FDA‐listed detergents, such as polysorbate‐20, while maintaining structural performance and cell viability (Jones et al. 2023).

From a safety and consumption perspective, the literature emphasizes three main aspects: (i) in vitro digestibility, with starch scaffolds showing adjustable degradability based on composition and protein‐based systems displaying rapid susceptibility to pepsin; (ii) cytocompatibility, assessed through leachate testing and direct culture assays; and (iii) the replacement of nonfood reagents with GRAS inputs and aqueous processing (Wang et al. 2024b; Wei et al. 2023; Wollschläger et al. 2022; Jones et al. 2023). Regarding allergenicity, although soy and pea proteins are established food ingredients, there is a lack of scaffold‐specific risk assessments. Dedicated immunoassays and appropriate labeling are therefore recommended when common allergens are used (Wollschläger et al. 2022; Bakhsh et al. 2024). Regulatory reviews further highlight that the use of approved additives and enzymes, combined with proper documentation of residuals, facilitates evaluation in the evolving framework for cell‐based foods (Levi et al. 2022; You et al. 2025).

As practical guidance, research groups should prioritize food‐grade inputs with traceable sourcing, such as GDL, calcium salts, and mTGase; adopt a standardized safety testing panel that includes cytotoxicity, simulated gastric digestion, and residual analyses; establish clear allergen management strategies; and implement documented process controls (washing, food‐approved detergents, residual monitoring). These practices strengthen the positioning of starch‐based scaffolds as edible materials, balancing cellular performance with safety requirements, and support regulatory dialogue in precommercial assessments (Levi et al. 2022; Jones et al. 2023; Bakhsh et al. 2024; You et al. 2025; Kumar et al. 2023).

## 5 Starch Modifications for Scaffolding Production

Native starches are attractive for scaffold fabrication due to their abundance, low cost, and ease of processing (Chen et al. 2024b). However, their native form often lacks sufficient mechanical and thermal resistance to endure demanding processing conditions, such as high temperatures, freeze–thaw cycles, exposure to acidic or alkaline environments, and intense shear forces (Bühler et al. 2022).

To enhance their performance, starches can be chemically (e.g., oxidation, esterification, etherification), physically (e.g., hydrothermal treatment, microwave processing, pregelatinization), or enzymatically modified, improving their physicochemical properties and expanding their functional versatility (Bühler et al. 2022). These strategies are often combined with the use of reinforcing agents or compatible polymers to overcome the inherent limitations of native starch. Despite these limitations, unmodified starches remain in use for specific applications, particularly when mixed with additives or other biopolymers that help balance their weaknesses and alter cell compatibility, as described in Table 2.

Another relevant example was presented by Shyam and Palaniappan (2024), who used commercial corn starch, obtained from SRL Chemicals (Mumbai, India), combined with gelatin to develop bioinks for 3D printing. This application reinforces the direct use of native starches in additive manufacturing technologies.

In porous scaffolds, commercial starches are also prominent. Soltani and Alizadeh (2023) produced bioactive scaffolds from starch combined with Aloe vera, 64S bioactive glass, and quail eggshell powder, highlighting the role of starch as a structural matrix. Beh et al. (2020) used commercial corn starch associated with nanohydroxyapatite (n‐HAp) to create porous scaffolds for bone regeneration. Mirab et al. (2018), in turn, combined commercial starch with PVA, citric acid, cellulose nanofibers, and nanohydroxyapatite particles, emphasizing its versatility as a base for structural composites.

More advanced studies have explored combinations of starches with cross‐linking agents and synthetic polymers. Aidun et al. (2018) modified medium molecular weight starch (342.30 g/mol), purchased from Sigma‐Aldrich (St. Louis, Missouri, USA), with 3‐glycidoxypropyltrimethoxysilane (GPTMS), creating porous scaffolds with greater structural stability and mechanical strength. Similarly, Jamshidi et al. (2019) combined starch, polycaprolactone (PCL), and montmorillonite nanoclays, developing hybrid scaffolds with optimized mechanical and bioactive properties.

Other applications include the use of starches in molded scaffolds and membranes. Long et al. 2024 used pharmaceutical‐grade corn starch, supplied by Aladdin Reagent Database Inc. (Shanghai, China), combined with silver nanoparticles and curcumin to manufacture molds with antimicrobial and antioxidant properties. In this formulation, starch acted as a biodegradable matrix that stabilized the active agents and provided structural integrity to the molded form.

Ceylan and Demir (2024) developed membranes from corn starch, obtained from Sigma‐Aldrich (USA), combined with polyvinyl alcohol (PVA), providing adequate structural support for tissue engineering applications. The incorporation of starch enhanced the biocompatibility and degradability of the membrane, while reducing reliance on synthetic polymers.

Additionally, Gomes et al. (2008) explored fibrous scaffolds made from starch and PCL, with promising applications in bone and cartilage regeneration. In this case, starch contributed to porosity and swelling behavior, improving nutrient diffusion and supporting cell infiltration during tissue remodeling.

### 5.1 Physical Methods

Physical modification techniques, such as heat‐moisture and ultrasonic treatment, enhance starch functionality without chemically altering its molecular structure. Physical modifications include thermal treatments like freezing, as well as more complex methods such as hydrothermal treatment, and nonthermal techniques that involve radiation, pressure, and ultrasonic treatments (Rashwan et al. 2024).

Physical methods, recognized for their more sustainable approaches, improve the structure–function relationships of starch (Lagunes‐Delgado et al. 2023; Mohammadzadeh and Milani 2020). Dual modification techniques and environmentally friendly methods are being explored to enhance the structural and functional characteristics of starch, making it suitable for advanced applications such as 3D printing and biodegradable packaging manufacturing (Lagunes‐Delgado et al. 2023; Raghunathan et al. 2021).

Freezing is an effective physical processing technique for modifying starch due to its mild and environmentally friendly conditions. During ultralow temperature freezing (−80°C), ice crystals formed can compress starch particles. This results in amylopectin leakage onto the starch particle surface, thereby increasing its water absorption capacity (Szymońska and Tomasik 2000). These structural changes in starch granules induced by freezing can affect the quality of starch‐based foods (Jia et al. 2023; Feng et al. 2024).

Wang et al. (2021) state that temperature variations significantly alter the organization of amorphous and crystalline regions in wheat starch, impacting its crystallinity and digestibility (Wang et al. 2021). Feng et al. (2024) show that exposure of starch to 230°C promotes internal changes in starch particles, whereas exposure to 80°C creates micropores on their surface. These thermal conditions modified the amylose and amylopectin composition of starch, improving viscosity stability and transforming slow‐digesting starches into resistant starches.

The combination of physical methods with other biopolymers is also viable. The addition of 4% pectin to corn starch improved its stability during freeze–thaw cycles and altered the starch retrogradation pattern (Zhang et al. 2023b). Heat and moisture treatment enhanced these modifications, reducing the degree of corn starch retrogradation to 39.7%, the syneresis rate to 6.33%, the double‐helix content to 26.5%, and the leached amylose content to 22.7%. This effect was due to the increased interaction between starch and pectin during the treatment, strengthening hydrogen bonds and resulting in a more stable starch structure less prone to retrogradation (Zhang et al. 2023b).

Chen et al. (2023a) found that the addition of NaCl/sucrose (a physical modulation via noncovalent solute–polymer interactions) delays starch gelatinization, particularly in starches with a high proportion of amylopectin chains and a loose granular structure. These cosolutes affect the viscoelasticity of starch, influencing the internal flexibility of amylopectin. The impact of NaCl/sucrose on starch retrogradation varies with the starch structure, cosolute concentration, and analytical method used. The retrogradation changes induced by cosolutes are strongly associated with the distribution of amylose chain length, where sucrose strengthens networks formed by short amylose chains, having less impact on chains that form stronger networks.

The use of atmospheric cold plasma, which utilizes air at normal pressure, has gained popularity for modifying starch granules due to its simplicity and low cost, eliminating the need for special gases or complex equipment. This technique has been applied to alter properties of various starch sources and has been compared with methods using special gases such as hexamethyldisiloxane, helium, argon, and nitrogen. Studies have also explored chemical and biochemical reactions to modify starch before or after cold plasma treatment, aiming to enhance its properties for specific applications (Leandro et al. 2023).

Gelatinization techniques have been applied to cassava and yam starches to adjust their physical and chemical characteristics. This method is used in the creation of edible films, where modifications in starch granule size and morphology can directly influence product functionality and quality (Ulyarti et al. 2021). In another study, corn, potato, and tapioca starches underwent a partial gelatinization process followed by freeze–thaw cycles with xanthan gum incorporation. This treatment improves the viscosity and stability of these starches, making them more suitable for a wide range of applications, from culinary to industrial uses (Chen and Lim 2021).

Maniglia et al. (2019) modified cassava starch through ozonation to assess its printability. With increased ozonation time, the starch exhibited higher carbonyl and carboxyl content, lower pH, and reduced molecular size, as well as gels with different strength behaviors depending on processing time. Starch ozonized for 30 min showed the lowest apparent peak viscosity and produced the strongest gel under all evaluated conditions. Both native starch gels and those ozonized for 30 min exhibited good printability at 65°C, but only the starch ozonized for 30 min maintained this property at that temperature (Maniglia et al. 2019).

Maniglia et al. (2020) applied DHT to modify cassava starch, resulting in a hydrogel with enhanced binding and textural properties, suitable for 3D food printing. This study highlights the relevance of adapting and refining processing techniques to expand the use of starches in new technological applications, including food manufacturing and bioactive materials (Maniglia et al. 2020).

When applied to scaffold fabrication, these modifications can enhance key properties such as porosity, mechanical stability, and cell adhesion. For instance, increasing surface area through micropores created by thermal treatments (Feng et al. 2024) can promote cell growth in porous scaffolds. Additionally, combining starches with others biopolymers or nanoparticles after physical modification, as observed in the studies by Zhang et al. (2023b) and Maniglia et al. (2020), demonstrates the potential of these techniques for creating functional, customizable, and sustainable scaffolds for tissue engineering applications. These advances reinforce starch's role as a promising and versatile material to meet the demands of tissue engineering and cultivated meat production.

### 5.2 Chemical Methods

The food industry has shown increasing interest in more sustainable methods, prioritizing green and consumer‐safe techniques over conventional chemical methods (Rashwan et al. 2024). Starch chemical modifications involve processes such as hydrolysis, etherification, esterification, cationization, crosslinking, oxidation, and graft copolymerization, as well as modification with octenyl succinic anhydride, adjusting the molecular structure of starch to enhance its properties (Gałkowska et al. 2023; Lewandowicz et al. 2022).

Acetylation, for example, stabilizes the rheological properties of starch, while crosslinking increases its thickening capacity, positively impacting texture and processing (Gałkowska et al. 2023). Cross‐linked and stabilized starches are widely used as food additives and have proven effective in improving the rheological and structural properties of potato starch (Lewandowicz et al. 2022).

These chemical alterations also influence the properties of aerogels, enhancing oil retention, emulsification, and stabilization (Le Thanh‐Blicharz et al. 2022). Sondari et al. (2021) demonstrated that cross‐linking cassava starch with sodium trimetaphosphate and alkaline catalysts resulted in significant changes in thermal analysis, morphology, and crystalline structure, making it more attractive for various applications.

Another example is acid‐treated starches, which are chemically modified through natural fermentation or lactic acid bacteria. This process improves expansion, solubility, thermal stability, and gelling potential, making them highly beneficial for various food applications (Bangar et al. 2022a). These properties are also relevant to cultivated meat production, as gelling capacity and structural stability are essential characteristics for scaffolds that support cell growth and maturation.

By correlating these chemical modifications with the production of scaffolds for cultivated meat, it becomes evident that techniques such as crosslinking, acetylation, and fermentation can enhance starch. Structural alterations induced by chemical modifications, as demonstrated by Sondari et al. (2021), can create scaffolds with greater water retention capacity and cell support, which are critical factors for the success of cultivated meat production.

Therefore, chemical modifications of starch offer a diverse set of tools to optimize its application in cultivated meat production. By adjusting specific properties such as porosity, strength, and bioactivity, these techniques make starch a highly versatile and promising material to meet the demands of this growing and innovative sector.

### 5.3 Enzymatic Methods

Enzymatic approaches to starch modification have gained prominence in the food industry, mainly due to their ability to reduce the formation of unwanted byproducts and coproducts, as well as their value in producing “clean‐label” starches with improved characteristics for various applications (Miao and BeMiller 2023).

These modifications involve the use of enzymes such as glycosyltransferases and glycosyl hydrolases, which act on starch hydrolysis and transfer reactions, improving its functional characteristics and nutritional value for industrial applications (Bensaad et al. 2022; Miao and BeMiller 2022). Additionally, specific enzymes such as branching enzymes and amylomaltases are essential for structuring starch and enhancing its functionalities (Bangar et al. 2022b).

One example is the dual enzymatic modification of corn starch, which significantly increases resistant starch content and improves structure, slow digestion properties, and gut health through the use of enzymes such as α‐amylase and branching enzyme (Liu et al. 2022; Li et al. 2022). These enzymatic modifications also alter granule morphology, crystalline type, paste properties, and swelling power, adding nutritional value to starch (Hutabarat and Stevensen 2023).

Enzymatic hydrolysis with pullulanase, for example, at 60°C in a pH 4 buffer solution, forms SNPs with great potential for use in packaging and medical applications (Chorfa et al. 2022). Different varieties of cassava starch also exhibit unique responses to enzymatic hydrolysis, allowing for specific customizations for various industries (Cornejo et al. 2022).

Additionally, amylosucrase, derived from *Deinococcus geothermalis*, used in the modification of potato starch, optimizes its digestive properties, making it more resistant to digestive enzymes, thus expanding its use in diverse applications (Jung et al. 2022).

These enzymatically optimized properties are directly applicable to the fabrication of scaffolds for cultivated meat. For example, SNPs obtained through enzymatic hydrolysis offer high surface area and controlled porosity, ideal characteristics for cell adhesion and proliferation (Cao et al. 2017). Resistant starches, in turn, ensure mechanical stability and structural functionality during cultivation (Guo et al. 2017), while the ability to customize starch properties through different enzymes allows for adjustments in specific properties such as water retention and viscosity (Moin et al. 2017), according to the production process demands. Thus, enzymatic methods present themselves as a sustainable and efficient solution for creating functional and adaptable scaffolds for cultivated meat production.

## 6 Potential Scalability of the Use of Starches for Scaffolding: Economic Aspects and Productivity

The challenges related to the scalability of starch use in cultivated meat production align with the broad applications and growing global production of this biopolymer (Mhaske et al. 2024; Kumar et al. 2023). While cultivated meat still faces critical bottlenecks, such as obtaining suitable cells, scaling up cell culture, developing serum‐free media, and constructing 3D tissue structures (Zheng et al. 2024b); the global starch market presents a favorable landscape to meet many of these technological demands (Table 3).

**TABLE 3:** Comparative analysis of production and economic feasibility of different types of starch for cultivated meat applications.

Properties	Corn	Potato	Cassava	Rice	Wheat
**Annual starch production—2022 (Million T)**	38.9 ^a^	1.25 ^a^	9.1 ^a^	1.3 ^a^	6.7 ^a^
**Crop yield (T/Ha)**	5.7 ^a^	21 ^a^	10.3 ^a^	4.7 ^a^	3.7 ^a^
**Starch percentage**	70% and 72% ^a^	12% and 20% ^a^	80% and 90% ^a^	70% and 80% ^a^	60% and 70% ^a^
**Gross starch yield (T/Ha)**	4.0 and 4.1 ^a^	2.5 and 4.2 ^a^	8.2 and 9.3 ^a^	3.3 and 3.7 ^a^	2.2 and 2.6 ^a^
**Cost (US$/T)**	350–400 ^b^	1140–1160 ^c^	450–500 ^a^	250–300 ^e^	600–800 ^d^
**Main producing countries**	USA, Canada, and Japan ^a^	Germany, Denmark, and Japan ^a^	Thailand, Vietnam, and Cambodia ^a^	Peru, Benin, and India ^a^	France, China, and Poland ^a^
**Potential production increase**	Expansion of cultivated areas ^a^	Genetic improvement ^e^	Production intensification ^f^	Expansion of cultivated areas ^g^	Expansion of cultivated areas ^a^

*Source*: Consolidated data from ^(a)^ FAO, ^(b)^ Procurement Tactics, ^(c)^ ChemAnalyst, ^(d)^ Clic Mercado, ^(e)^ PotatoPro, ^(f)^ CassavaNet^g^, and ^(g)^International Grains Council.

The annual global production of starch exceeds 50 million tons, with corn, rice, cassava, wheat, and potato as the main sources (Rashwan et al. 2024). This abundance reinforces starch's strategic position, not only in food and bioplastics but also in innovative applications such as scaffolds for cultivated meat. The growth of the modified starch market and the expansion of bioplastics, projected at 20% annual growth, further support this trajectory.

In 2021, the United States commercialized 33.4 million metric tons of starch (27% of global volume). Projections show China reaching 33.1 million metric tons by 2026, with an annual growth rate of 8.1%. Europe, led by Germany, is expected to reach 35.7 million metric tons, with varied growth rates across countries (GlobeNewswire 2021). These projections reflect increasing industrial reliance on starch and its derivatives.

Among starch sources, cassava stands out, particularly in developing countries like Nigeria and India, accounting for 35.26% of starch‐rich root and tuber production (Mukhametzyanov et al. 2021). In Brazil, cassava starch production reached 676.7 thousand tons in 2023, an increase of 29% compared to the previous year, driven by a higher volume of cassava root processed and greater starch content (Table 3). The production of modified starches in the same period was 94.9 thousand tons (CEPEA/ESALQ 2023), demonstrating growing interest in customized starch applications.

However, its adoption in this emerging sector will depend on which current applications it displaces. If starch is already used efficiently with minimal waste in existing industries such as food, bioenergy, or packaging, diverting it to cultivated meat scaffolds may raise competition and sustainability concerns, similar to debates around the use of food crops for biofuels (Godfray et al. 2010).

Comparative data in Table 3 highlight the economic viability of different starch types for scaffold fabrication. Corn starch leads in annual production (38.9 million tons), supported by an efficient global supply chain. Potato starch, while offering the highest productivity per hectare (21 tons), has lower starch content, limiting its industrial efficiency (FAO. FAOSTAT 2024). Cassava starch combines high starch percentage (80%–90%) with solid productivity (8.2–9.3 t/ha), making it highly efficient in land use. Meanwhile, rice starch offers the lowest production cost ($250–300/ton), which is particularly attractive for reducing scaffold fabrication costs (Table 3).

To ensure scalability and sustainability, strategies such as expanding cultivated areas and improving cultivars through genetic techniques are under exploration (FAO. FAOSTAT 2024; PotatoPro 2024; CassavaNet 2024). These advances enhance starch yield and consistency, paving the way for its integration into advanced scaffold technologies, including hydrogels and aerogels, which require tailored hydration capacity, porosity, and mechanical properties for 3D cell culture.

In Brazil, data from Table 3 show that corn produced 64.8 thousand tons of starch in 2022 with a yield of 5.2 t/ha, resulting in 3.6–3.7 t/ha of gross starch productivity. Cassava, in the same year, produced 46.2 thousand tons from a yield of 14.9 t/ha, reaching up to 13.4 t/ha in gross productivity (FAO. FAOSTAT 2024). These numbers position cassava as the most efficient crop in terms of starch yield per hectare under Brazilian conditions. Building on these comparisons, cassava starch stands out as an attractive candidate for scaffold production in cultivated meat when cost, availability, and land efficiency are considered. Nevertheless, rice and corn starches remain viable alternatives due to their affordability and global supply. The ultimate choice, however, must also account for compatibility with scaffold fabrication techniques and the requirements of the cell culture environment.

Beyond agricultural yield and supply, scalability depends on the successful integration of starch‐based scaffolds into bioprocesses already applied to cultivated meat production (Pajčin et al. 2022; Zo et al. 2025; Gome et al. 2025). In suspension bioreactors, starch‐based hydrogels or microparticles can act as carriers for cell expansion, while fixed‐bed and perfusion systems allow the use of porous starch matrices to support 3D tissue formation (Norris et al. 2022; Hasebe et al. 2023; Lee and Hwang 2023). As illustrated in Figure 1A, the conceptual flowchart of cultivated meat production highlights these integration points: the process involves cell isolation, proliferation in suspension bioreactors, and differentiation within scaffolds, where starch‐based matrices serve both as carriers for expansion and as structural supports for tissue maturation. The feasibility of this approach is closely tied to the development of food‐grade cross‐linking strategies and reproducible rheological control, both essential for automated handling and large‐scale manufacturing (Kawecki et al. 2024).

Although starch is an inexpensive agricultural raw material, its application in cultivated meat introduces additional costs linked to industrial processing. Converting starch into hydrogels, aerogels, or bioinks with reproducible rheological properties requires modification, crosslinking, and purity control steps that significantly raise the final scaffold cost compared with the raw material price (Sardelli et al. 2021; Barrulas and Corvo 2023; Kumar et al. 2025). Competition with established markets, such as biodegradable packaging and bioenergy, further complicates scalability, as these sectors already consume increasing volumes of modified starch (Bergel et al. 2020; La Fuente et al. 2019; Xu et al. 2023). Large‐scale economic feasibility will therefore depend on developing optimized industrial processes that can reduce transformation costs and ensure batch‐to‐batch uniformity, an essential requirement for integration into complex bioprocesses (Kawecki et al. 2024). These considerations will determine whether starch scaffolds remain confined to laboratory studies or progress toward effective use in industrial cultivated meat systems.

## 7 Conclusion

Starch‐based scaffolds stand out as a promising and sustainable option for cultivated meat, offering advantages such as edibility, abundance, and cost‐effectiveness. Their functionality, however, depends largely on modifications or hybrid formulations, since native starch alone rarely provides the mechanical strength, stability, and adhesive properties required for efficient cell growth and tissue maturation. The distinctive contribution of this review lies in emphasizing food‐grade applicability. By addressing safety, digestibility, and regulatory aspects in addition to biological performance, it bridges biomedical knowledge with the specific requirements of edible systems for cultivated meat.

Key challenges remain, including control of degradation rate, batch‐to‐batch variability, competition with established starch markets, and the validation of food‐grade crosslinkers and additives. Overcoming these barriers will be essential to move starch scaffolds from laboratory‐scale prototypes to industrial applications. If successful, starch may consolidate its role as a versatile and accessible biopolymer for sustainable meat biomanufacturing.

## Author Contributions

**Cristiane Silvano Wensing**: investigation, writing—original draft, methodology, formal analysis, data curation. **German Ayala Valencia**: writing—review and editing, validation, methodology, supervision. **Edna Regina Amante**: writing—review and editing, validation, methodology, visualization. **Mark Post**: validation, visualization, writing—review and editing, methodology. **Silvani Verruck**: conceptualization, funding acquisition, project administration, writing—review and editing, validation, supervision, resources.

## Conflicts of Interest

Mark Post is a cofounder and stakeholder of Mosa Meat B.V; a company aiming to commercialize cultivated meat technologies, at the time of writing. Silvani Verruck is a visiting researcher at Mosa Meat B.V. The other authors declare no conflicts of interest.
